# How the Motility Pattern of Bacteria Affects Their Dispersal and Chemotaxis

**DOI:** 10.1371/journal.pone.0081936

**Published:** 2013-12-31

**Authors:** Johannes Taktikos, Holger Stark, Vasily Zaburdaev

**Affiliations:** 1 Max-Planck-Institut für Physik komplexer Systeme, Dresden, Germany; 2 Technische Universität Berlin, Institut für Theoretische Physik, Berlin, Germany; 3 Harvard University, School of Engineering and Applied Sciences, Cambridge, Massachusetts, United States; University of California Irvine, United States of America

## Abstract

Most bacteria at certain stages of their life cycle are able to move actively; they can swim in a liquid or crawl on various surfaces. A typical path of the moving cell often resembles the trajectory of a random walk. However, bacteria are capable of modifying their apparently random motion in response to changing environmental conditions. As a result, bacteria can migrate towards the source of nutrients or away from harmful chemicals. Surprisingly, many bacterial species that were studied have several distinct motility patterns, which can be theoretically modeled by a unifying random walk approach. We use this approach to quantify the process of cell dispersal in a homogeneous environment and show how the bacterial drift velocity towards the source of attracting chemicals is affected by the motility pattern of the bacteria. Our results open up the possibility of accessing additional information about the intrinsic response of the cells using macroscopic observations of bacteria moving in inhomogeneous environments.

## Introduction

Bacteria constitute a major part of the biomass on our planet [1]. They come in different shapes and sizes and are able to swim in water and crawl on surfaces [2]. Bacteria build complex colonies called biofilms [3] and find ways to adapt to the harshest environmental conditions [4]. One of the ways cells react to changes in the environment is by employing various “taxis-strategies”. In response to gradients in temperature, chemicals, or electric fields [5], bacteria are able to alternate their motility to locate favorable niches and avoid dangerous locations. Chemotaxis is one of the best studied examples of this behavior and its biochemical mechanisms in bacteria are rather well understood [6]. However, bacteria moving in homogeneous environments often have a very distinct motility pattern, which is defined by the phenotype of the cell. It remains unclear how different motility patterns of bacteria can affect their ability to perform chemotaxis. In this paper, we propose a generalized random walk description of a broad class of observed bacterial motility patterns. It allows us to describe quantitatively the dispersal process of bacteria and calculate the effect of the motility pattern on the chemotactic behavior of the cells. This rigorous description creates the possibility of accessing additional information about the intrinsic response of the cells using macroscopic observations of bacteria moving in constant gradients or towards the point source of a chemical.

The run-and-tumble motion of *E. coli* bacteria is probably the best-known example of bacterial swimming. *E. coli* has multiple flagella, which can rotate and propel the cell forward. Flagella rotating in the counterclockwise (CCW) direction form a bundle and the cell is in the “run” mode of highly persistent motion. When one or several flagella reverse the direction of rotation, the bundle comes apart and the cell body performs an irregular tumbling motion [7]. Usually, there is little displacement during the “tumbling” mode and it mainly serves to reorient the direction of the cell for the next run. For *E. coli*, the turning angles are randomly distributed with an average of about 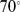. Many marine bacteria, such as *S. putrefaciens* or *P. haloplanktis* [8], that have just a single flagellum simply reverse the direction of their swimming when the flagellum switches the direction of rotation; this results in a turning angle distribution peaked around 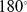. Interestingly, the run-reverse strategy is not exclusive to swimming cells but was also observed for bacteria moving on surfaces. Some bacteria, as for example *M. xanthus* [9], [10], can also use different cell appendages called pili [11], [12] or even more complex mechanisms, to attach to and actively move on surfaces. In this case, the alternation of pili activity on different poles of elongated cells also leads to the run-reverse motility pattern.

In response to changing environmental conditions, like a difference in concentration of a certain signaling chemical or nutrient, bacteria are able to regulate the durations of their run phases [13]. On average, runs become longer if a bacterium moves towards the source of the attracting signal and shortened if it moves away from the source [5], [14]. It is important that in bacteria the probability to tumble or to continue a run depends on the concentration of the chemical sampled by the cell during its motion for a certain time interval, weighted by the internal response function of the cell [15]. Therefore, the chemotactic behavior and the motility pattern of bacteria are tightly coupled together. Recently, another pattern of swimming was reported for *V. alginolyticus* bacteria [16]. These marine bacteria also have one flagellum, but during each second reversal its rotation is unstable and leads to a random turn of the cell body [17], [18]. Hence, a trajectory of these bacteria is composed of strictly alternating 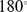 reversals and random turns with an average of 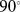. Remarkably, *V. alginolyticus* were three times faster in gathering around the source of a chemoattractant when compared to *E. coli* [16], [19]. To test if such an increased performance during chemotaxis can be attributed to their peculiar motility pattern, we developed a random walk model describing the trajectories of bacteria. It allowed us to calculate analytically the diffusion constants in the absence of the chemical and the drift speeds in a small linear gradient of chemoattractant. In particular, we show that the motility pattern alone cannot explain the experimentally observed difference between the chemotactic behavior of *V. alginolyticus* and *E. coli*. This strongly suggests that, instead, a difference in the response functions of the bacteria is the key feature that leads to the distinct behaviors observed experimentally. Our model can serve as an analytical tool to test for various response strategies of individual cells and relate them to the observed macroscopic agglomeration dynamics.

### Motility patterns

We start with a brief description of three distinct motility patterns exhibited by bacteria. It appears that the motility of quite a large part of studied or practically relevant bacterial species can be attributed to one of these three classes. We first focus on a two-dimensional setup, since many tracking experiments for swimming cells are performed in planar geometry and surface-related motility is naturally two-dimensional. We will however show how to generalize our results to higher dimensions.

**Figure 1:**
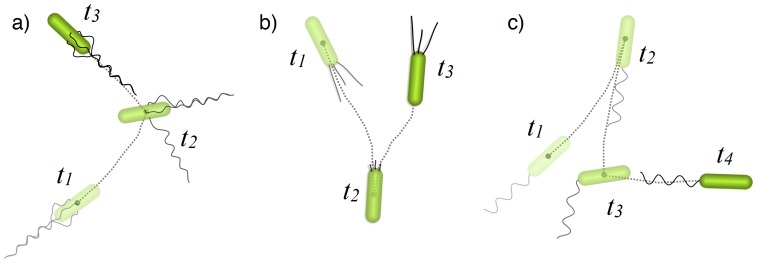
Sketch of the predominant motility patterns. a) Run-and-tumble, b) Run-reverse, and c) Run-reverse-flick. During a “run” event, a cell moves with high persistence. Runs are interrupted by reorientation events like tumbling or reversal. The time steps 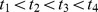 indicate the sequence of these events. An average turning angle after tumbling in *E. coli* bacteria is 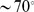 (a), whereas it is an almost perfect reversal of 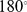 for many marine bacteria, or cells with twitching motility due to cell appendages, called pili (b). *V. alginolyticus* (c) alternates reversals (at 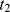) with randomizing flicks (at 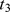) with an average turning angle of 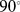.

Swimming *E. coli* alternate persistent runs with tumbling events (see Fig. 1a). The duration of tumbles on average is about ten times shorter than the duration of runs, and in our model we will assume this time to be vanishingly small (however, see also Ref. [20], where tumbling times were explicitly modeled). The distribution of run times is well approximated by the exponential function with a mean value of 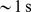 [13]. Recent experiments on tethered cells and accompanying theoretical analysis also suggest the possibility of run times with a power-law distribution [21], [22]. Each run does not follow a perfectly straight line. The interaction of the cell body and flagella with the surrounding fluid results in a fluctuating direction of the cell velocity, which can be well described by rotational diffusion [13]. The speed of the cell during a single run and from one run to another is nearly constant [14], [23]. Depending on the environmental conditions, the typical speed of *E. coli* is in the range of 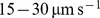 [13], [24]. After a tumbling event, the new direction of swimming has on average an angle of 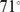 with the direction of the previous run [13].

Up to 70% of marine bacteria [25] and also bacteria twitching on surfaces, such as *P. aeruginosa* or *M. xanthus*, adopt a similar strategy to that of *E. coli*, but with 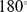 reorientation events (see Fig. 1b). The speed of their forward and backward motion is usually comparable [26]. Note that the run speeds of marine bacteria can reach up to 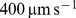 [27], whereas cells twitching on a surface are much slower with typical speeds of 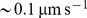 [28]. The motility pattern of another marine bacterium, *V. alginolyticus*, is similar to the run-reverse strategy. However, the flagellum of these cells is unstable when its rotation switches from CW to CCW direction, leading to the appearance of “flicks” – completely randomizing turning angles with an average of 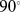 (see Fig. 1c) [16]. Durations and speeds of runs after reversal or flick are fairly similar [16].

## Analysis

**Figure 2:**
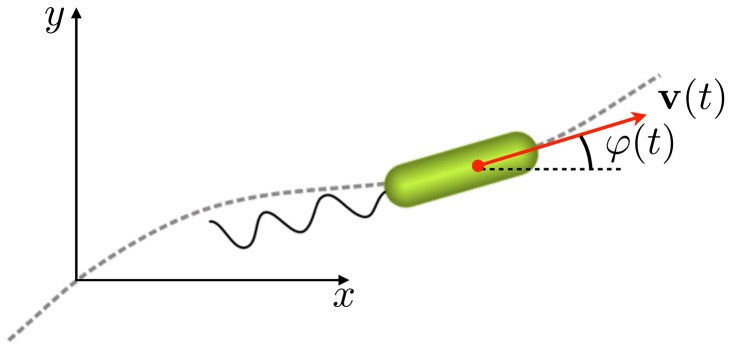
Setup of the model. A cell with velocity 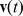 moves at constant speed 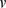. The angle 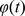 between the velocity vector 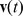 and the 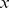 axis defines the direction of cell motion.

To describe quantitatively the dynamics of dispersal of the bacteria exhibiting the above motility patterns, we propose the following generalized random walk model. Each random walker representing a single bacterium moves with velocity 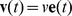, where the speed 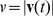 is constant and the unit vector 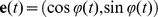 denotes the direction of propagation at time 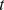, see Fig. 2. Integration of the velocity with respect to time yields the particle's trajectory 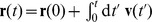. It will be our first goal to determine the velocity autocorrelation function
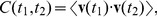
(1)where 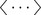 denotes the ensemble average. It is directly connected to the mean squared displacement (MSD) via the Kubo relation
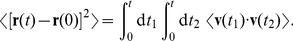
(2)If the MSD is a linear function of 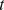 for large times, the diffusion coefficient can be defined as 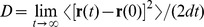, where 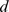 is the spatial dimension [29], [30]. Durations of runs are random and described by the probability density function (PDF) 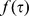. For the model with two types of events we will allow for two separate PDFs of the run time after the corresponding reversal (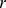) or flick (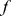) event, 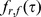. When a run is interrupted by a turning event (tumbling or reserval), the particle's direction of motion changes instantaneously by an angle 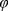, drawn from the probability density
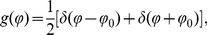
(3)where 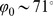 for run-and-tumble of *E. coli* and 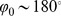 for run-reverse motion. Note that assuming a delta-peaked distribution for 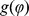 is a minor simplification; as we also show in Sec. III of Text S1, our results do not change if one considers a continuous distribution which leads to the same persistence parameter 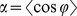. The turning angles for run-reverse and flick mode will be alternatingly chosen as 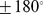 (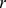) and 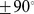 (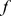).

In the case of constant speed, the correlation function 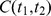 is determined by the dynamics of the angle 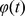 describing the direction of the cell's motion,
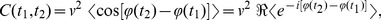
(4)where 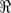 denotes the real part; note that, for symmetry reasons, the imaginary part vanishes after averaging. The random walk dynamics of the angle 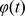 can be decomposed into two parts,
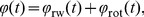
(5)where 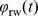 models the actual random walk due to a specific motility pattern with straight paths and jumps in the angle given by Eq. (3), and 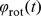 describes angular changes due to rotational diffusion. It is natural to assume that the effects of fluctuations during the runs are independent of the reorientation events resulting from tumbles and reversals. Therefore, the averaging in Eq. (4) can be decoupled into
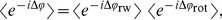
(6)where 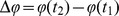. The velocity correlation function factorizes into a pattern-specific part 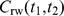 and a factor due to rotational diffusion 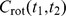:
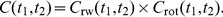
(7)The latter is known to be 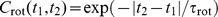, where 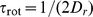 is the characteristic rotational diffusion time showing how fast a particle is forgetting its direction of motion, and 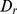 is the rotational diffusion constant [31]. The averaging of the random walk part 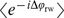 from Eq. (6) can be expressed as
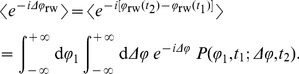
(8)Here, 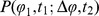 is the joint probability density to find a particle with direction 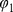 at time 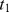 and direction 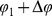 at time 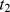. We define the Fourier transform of a function 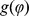 as 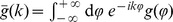, and observe that Eq. (8) corresponds to a double Fourier transform of 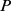 with respect to 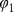 and 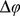, where the corresponding coordinates in Fourier space are set to 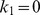 and 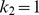, respectively:
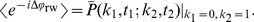
(9)To find the joint PDF 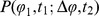, we note that it is just a two-point density for a continuous time random walk model (CTRW) [32]–[34], where the angle 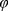 performs this random walk. We now show how to solve the problem for the three motility patterns in question.

## Results

### Random walk with one turning angle

For run-and-tumble and run-reverse motion, the angular jump distribution 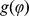 is given by Eq. (3). In this case, we make use of a result from random walk theory for the joint probability 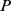 entering Eqs. (8, 9) [32]–[34] (see Sec. I of Text S1 for details). To proceed, we define the Laplace transform of a function 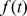 as 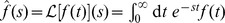; the combined Fourier-Laplace transform of a function 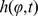 is denoted as 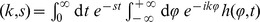, where the Laplace transform corresponds to the variables 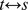 and the Fourier transform corresponds to 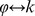. After introducing the survival probability 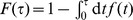, one obtains
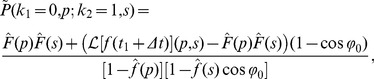
(10)where the correspondence 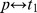 and 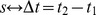 applies for the Laplace transform; note that 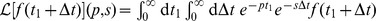 can be rewritten as 
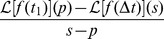
, see for instance Ref. [35]. This is a very general result for the two-point density function, where the evolution for 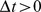 depends on the pre-history of the system until time 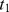. It is therefore capable of describing processes with aging, in particular with run times, which follow a power-law distribution [32]–[34]. Formally, we have thus solved our random walk model analytically for any distribution 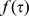 of run times. To calculate the correlation function 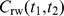, one has to find the inverse Laplace transform of Eq. (10) with respect to 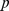 and 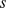, and this sometimes presents a technical difficulty. To apply our result to relevant biological examples, we will focus on two special cases, namely run times which follow an exponential or power-law distribution.

### Exponential distribution of run times

**Figure 3:**
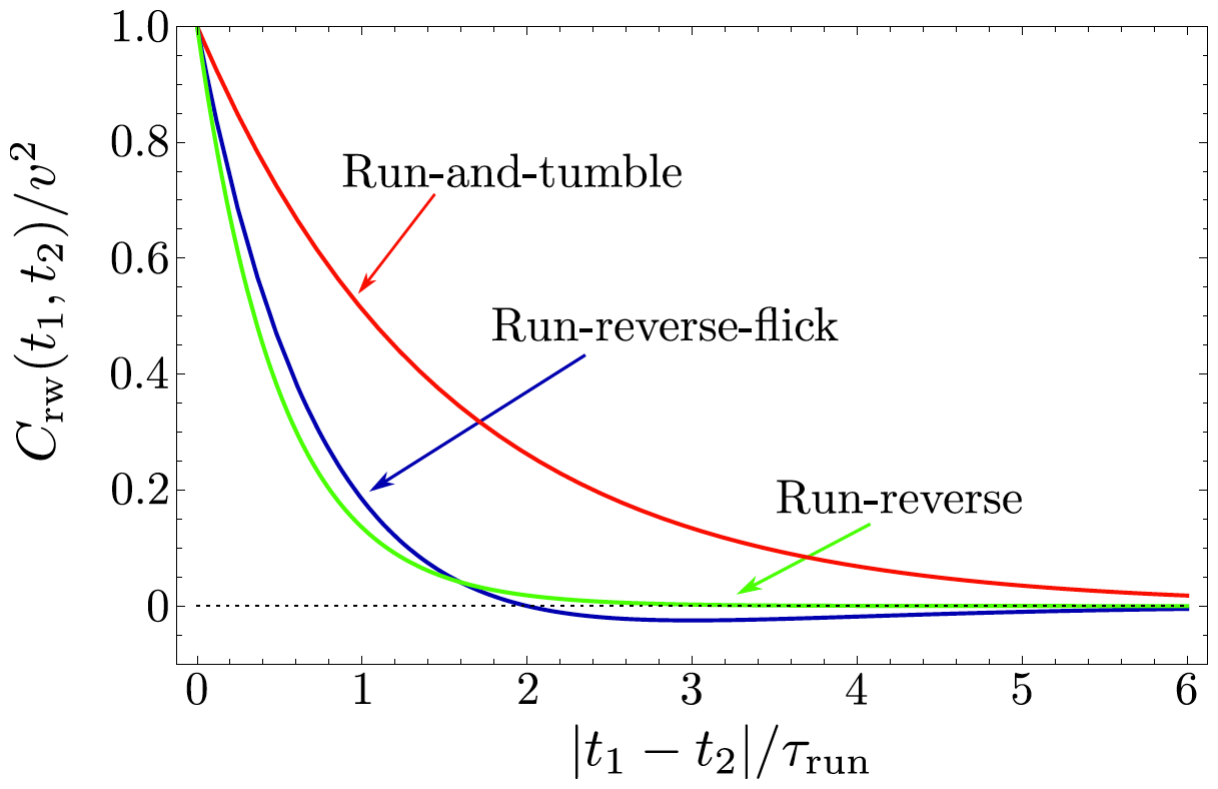
Velocity correlation function. The normalized velocity correlation function 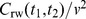 is plotted as a function of dimensionless time 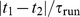. The curves are shown for run-and-tumble of *E. coli* with persistence parameter 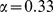 (red), run-reverse with 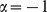 (green), and run-reverse-flick with alternating 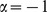 and 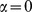 (blue). The analytical expressions are given in Eqs. (12) and (21), respectively.

**Figure 4:**
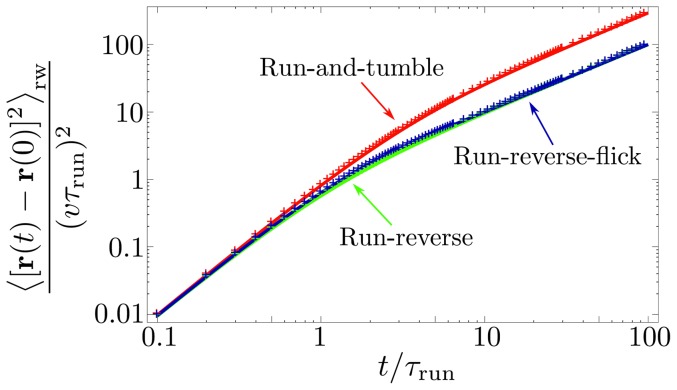
Mean squared displacement (MSD). The curves of the normalized MSD versus dimensionless time 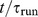 correspond to *E. coli*'s run-and-tumble with 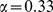 (red), run-reverse with 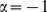 (green), and run-reverse-flick with alternating 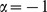 and 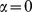 (blue). The analytical expressions are given in Eqs. (13) and (22), respectively. The crosses are obtained from numerical simulations and fully agree with the analytical results.

We first consider an exponential run time distribution,
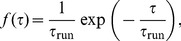
(11)where 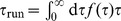 is the mean run time. Since the exponential PDF is the memoryless distribution, Eq. (10) simplifies considerably. Performing two inverse Laplace transforms and using Eqs. (4, 8), we obtain a well-known result for the velocity autocorrelation function [36],
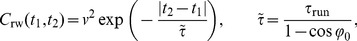
(12)which decays exponentially on the time scale 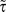. It is plotted for run-and-tumble motion for *E. coli* (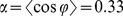) and run-reverse (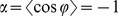) in Fig. 3. With the help of Eq. (2), we find the MSD for the random walk pattern (without rotational diffusion),
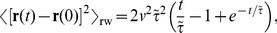
(13)whose analytical form also arises from the Ornstein-Uhlenbeck process of a Brownian particle [37]. Note that, up to this point, our results were derived for the model in 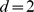. In Sec. III of Text S1, we show that Eq. (13) is also valid for 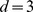. Therefore, below we use 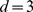 to compute the diffusion constant and compare with known results. For small times 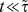, the MSD from Eq. (13) describes ballistic motion; for large times 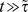, the MSD scales linearly in time, 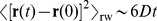 (see Fig. 4), with diffusion coeffficient [13], [36]

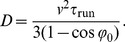
(14)Note that the regime of reversals, where cells backtrack along their previous path, has a minimal diffusion constant, which is two times smaller than in the case of completely random reorientations with 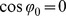. The limiting case of 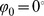 generates ballistic motion, such that 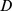 diverges. However, this divergence can be regularized by rotational diffusion during the run events. As a consequence of Eq. (7), the full velocity autocorrelation function then becomes
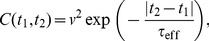
(15)which gives rise to the characteristic time scale 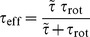, or
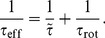
(16)


### Power-law distribution of run times

In Refs. [21], [22], it was pointed out that cells of *E. coli* can have power-law distributed run times,
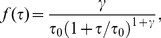
(17)with exponent 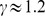. The power-law distribution (17) leads to a finite mean run time 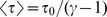 (for 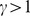), but the average of the squared run length diverges, leading to anomalous diffusion [35]. Also because of the power-law tail in the distribution of run times, memory effects play a significant role in the transport process. This means that the probability that a cell tumbles within a small time interval depends on its history. However, with the help of the general expression Eq. (10), which explicitly takes into account these memory effects, we can calculate 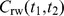. The double inverse Laplace transform required to compute the correlation function in the real time domain presents a technical challenge, which can be resolved numerically. Analytically, it is possible to consider the asymptotic behavior of the correlation function for large 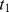 and 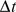 (corresponding to the limit 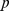, 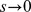 in Laplace space). An asymptotic analysis for 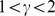 leads to
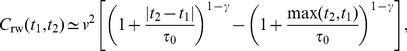
(18)valid for large 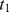, 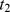, and 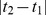. One of the remarkable results here is that in the asymptotic regime the correlation function and therefore the MSD do not depend on the turning angle distribution: Long persistent runs dominate over geometric effects. From Eq. (18), it follows that the MSD displays superdiffusive behavior for large times, when the MSD grows faster than linearly in time: 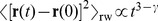. In fact, the trajectories of bacteria in this regime represent a two-dimensional realization of a Lévy walk [35], [38]. If we now consider rotational diffusion during the runs it makes the dispersal normal again and for large times the MSD scales linearly in time.

### Random walk with alternating turning angles: Run-reverse-flick

We now discuss the motility pattern, which is represented by the alternation of a forward run, reversal event, backward run, and flick event. The angular jump distribution from Eq. (3) is thus different for reversal and flick angles,
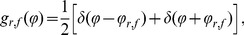
(19)with 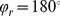 and 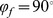. We also allow for two different distributions for run times after reversals 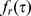 and after flicks 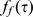.

To determine the joint probability density 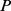, we formulate and solve the full set of equations of the underlying CTRW for the direction 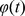. In Sec. I of Text S1, we sketch the derivation and present our analytical result for the two-point PDF 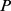. It is exact and holds for arbitrary run time and turning angle distributions in its most general form. In the following, we restrict our study to the experimentally relevant case of exponential distributions, as given in Eq. (11), but we allow for two different mean values 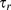 and 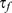, corresponding to run times after reversal and flick events, respectively. Our approach yields an exact analytical result for the velocity autocorrelation function,
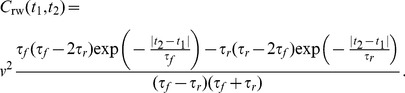
(20)For *V. alginolyticus*, the mean run times are similar with 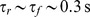. For a single run time 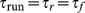, Eq. (20) then reduces to
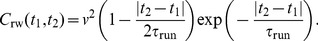
(21)A peculiar feature of this correlation function is that it becomes negative for 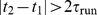, see also Fig. 3. Note that for the run-reverse pattern without flicks, the correlations are always positive, see Eq. (12). Next, we use Eq. (21) to obtain the expression for the MSD,
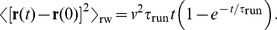
(22)The functional form of this MSD is different from the corresponding expression for the random walk with a single turning angle [Eq. (13)]. However, it is striking that the resulting diffusion coefficient 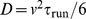 is identical for run-reverse and run-reverse-flick motion, see Fig. 4. This degeneracy vanishes if rotational diffusion during the runs is taken into account; the diffusion coefficient then reads (for 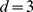)
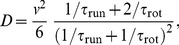
(23)and, in general, 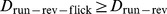. It is instructive to present the result for the diffusion coefficient with 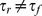 (for details, see Sec. I of Text S1):
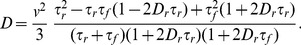
(24)The exact answers (23) and (24) differ from previous simple estimates [16], [19], [39]. For example, an intuitive attempt to use an average value of 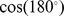 and 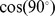 and substitute it into Eq. (14) [19] yields an incorrect result. We also see that the diffusion constant does not vanish when 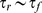, cf. [16].

#### Comparison of the diffusion coefficient for *E. coli* and *V. alginolyticus*

As typical parameters, we set 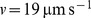 and 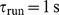 for *E. coli*, and 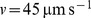 and 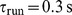 for *V. alginolyticus* [13], [16], [24], [40]. A realistic rotational diffusion constant, which is applicable to both types of bacteria, is 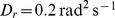 [16], [41]. With these numbers, the diffusion coefficients (in 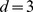) read
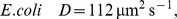


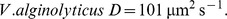
Note that the diffusion coefficient is sensitive to the rotational diffusion constant 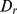 if the mean run time is comparable to the time scale of rotational diffusion 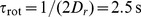, as is the case for *E. coli*. For example, the diffusion coefficient of *E. coli* becomes 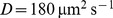, if we neglect rotational diffusion and set 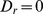.

This demonstrates how the rigorous theoretical model can quantitatively describe the dispersal of bacteria in a homogeneous environment. We next investigate the process of chemotaxis.

### Chemotaxis

If a bacterium, such as *E. coli*, is exposed to a gradient of chemoattractant, for example amino acids or sugars, with concentration 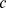, it changes its unbiased run-and-tumble strategy in order to move along the gradient [13]. To do so, the genetic chemotactic pathway of *E. coli* extends the run times if the cell swims in the direction of increasing concentration 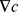 [14]. The bacterial response to a short pulse of chemoattractant is measured by the fraction of time that a flagellum tethered to a surface rotates CCW [42]; the response reveals a biphasic behavior. After the stimulation with the chemical pulse, the fraction quickly reaches a maximum and remains above the baseline for 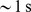, then it falls below and finally approaches the baseline after 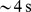 [15]. The shape of this curve for the fraction of CCW rotation motivated the introduction of a response function 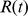. In Ref. [43], the response function was used to linearly connect the tumbling rate 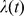 of a bacterium to the concentration of chemicals it experienced along its path,
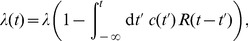
(25)where 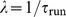 is the cell's tumbling rate in a homogeneous environment. In fact, 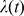 hardly increases when a cell moves against the gradient [14], [15]; however, we use the full expression from Eq. (25) and note that our calculations could also be modified to account for this detail. In this paper, we employ the following analytical expression for the response function, which was frequently used in previous work [41], [44], [45]:
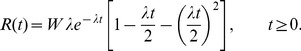
(26)Here, we introduce a single normalization constant 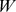 with the dimension of volume. One of the interesting features of 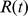 is that it satisfies the adaptive response property 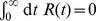.

In Ref. [46], de Gennes used Eq. (25) and a perturbation theory approach to calculate the chemotactic drift velocity of bacteria, 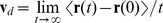, in the presence of a small gradient 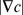. This result was generalized by Locsei for *E. coli* by including the directional persistence between tumbling events and rotational diffusion during runs [45]. Note that the result is fully consistent with the different approach by Celani and Vergassola from Ref. [41]: The authors also assume the tumbling rate from Eq. (25), but they introduce additional Markovian internal variables and arrive at a Fokker-Planck description. The hydrodynamic limit provides expressions for the chemotactic sensitivity 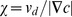 and bacterial diffusivity 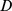. The directionality parameter 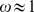 from [41] corresponds to our persistence parameter 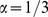
—the value of *E. coli*—, and using the adaptive response property, both the diffusion constant and chemotactic sensitivity from [41] agree with [13], [36], [45].

**Figure 5:**
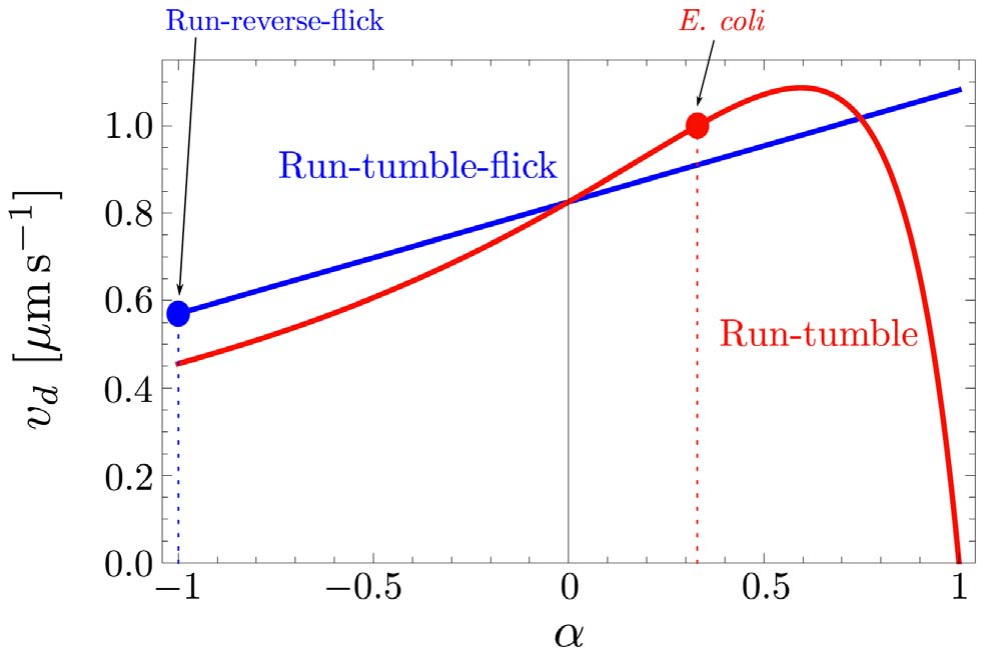
Comparison of the chemotactic drift speed 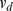 versus persistence parameter 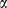 between run-tumble-flick [Eq. (28)] and run-tumble [Eq. (27)]. All parameters are adjusted to *E. coli* in the gradient 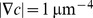 with 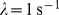, 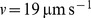, 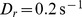, and 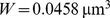.

Using the response function from Eq. (26), the drift speed 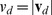 is given by [45]

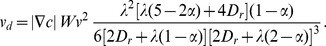
(27)This is plotted as a function of 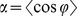 in Fig. 5 (red curve). We are primarily interested in the effect of the motility pattern on the chemotactic drift speed 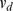. Therefore, we use the chemotactic response function of *E. coli* for our modeling; to the best of our knowledge, it is also the only experimentally measured one. However, we recall that the chemotactic response of bacteria, such as *E. coli*, *B. subtilis*, or *R. sphaeroides*, has been recently modeled on a biochemical level [47], [48].

### Chemotactic drift speed for run-tumble-flick motion

We want to compare the chemotactic drift speed of run-and-tumble with persistence parameter 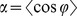 to a random walk, where one turn happens with the same 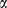, but every second angular change stems from a flick, which destroys any directional persistence. We denote the second pattern as “run-tumble-flick”; setting 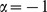 yields the run-reverse-flick strategy.

Now it is important to consider the motion of cells in three dimensions. We assume a small chemical gradient 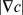 in the 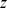 direction, and the concentration 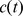, which is experienced by the bacterium at position 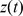, becomes 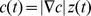. In the simplest case, the times for runs after tumbles and flicks are equally distributed with mean 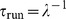. We then take the approach from Eq. (25) and perform a calculation of 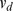 in the spirit of Refs. [45], [46]: To first order in 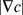, we determine the mean displacement during a forward and subsequent backward run, 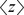, which yields the chemotactic drift speed 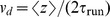. We arrive at the following result (see Ref. [49] and Sec. IV of Text S1 for details):
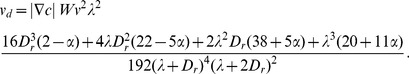
(28)The chemotactic drift speed 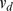 is always positive and a linear function of 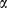 (see the blue curve in Fig. 5). The red curve of Fig. 5 shows 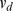 for the same parameters but without flicks. For negative persistence parameter 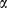, the additional flick event helps the random walker to approach the gradient better, and 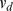 is larger with a flick for 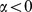. For 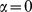, both random walk processes are equal as they have no persistence, and the curves intersect. For increasing 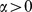, 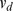 is smaller in the presence of the randomizing flick event. Finally, there is a pronounced difference at 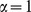, where 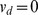 for the run-and-tumble strategy, while 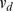 becomes maximal for run-tumble-flick motion. This is easy to understand since the 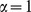 in the run-and-tumble model entails no turning events for the cell and the cell is not able to move actively in the direction of the gradient. An additional flick clearly allows the cell to reorient.

Note that a similar calculation for the run-reverse-flick pattern is also found in Ref. [39], where the chemotactic drift is determined only for the delta-response 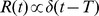 and without rotational diffusion. Our result in Eq. (28) is based on the response function of *E. coli* and explicitly shows the influence of rotational diffusion on the chemotactic drift speed.

#### Comparison of the chemotactic drift speed for *E. coli* and *V. alginolyticus*

In recent experimental work [24], the chemotactic drift velocity of *E. coli* in a constant gradient of the amino acid serine was measured. It is important to note that the perturbative nature of the analysis we used to calculate the drift velocity assumes a very small gradient. An obvious limitation on the gradient arises from Eq. (25), where the rate 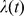 cannot become negative. Therefore, we use the value of the drift speed for the smallest measured gradient (see Sec. II of Text S1) and formula (27) to calculate the only remaining unknown parameter 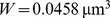. As before, we use 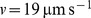, 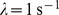 for *E. coli*, and 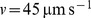, 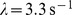 for *V. alginolyticus*. Rotational diffusion is set to 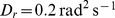, and the value of the gradient is 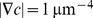. Finally, we stress that we choose the same shape and prefactor 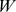 of the response function for both bacteria.

**Figure 6:**
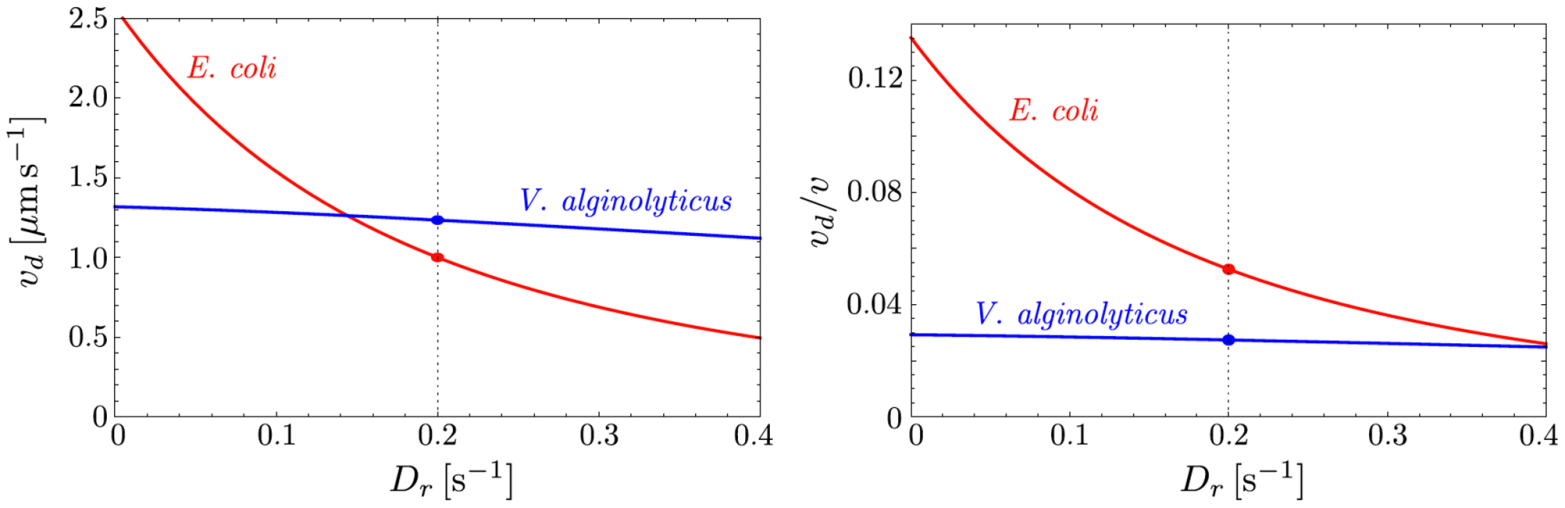
Chemotactic drift speed as a function of 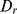 for *E. coli* and *V. alginolyticus*. The plot on the left shows 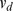; on the right, the chemotactic drift is normalized by the swimming speed as 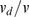 and coincides with the chemotactic index.

For *E. coli*, the chemotactic drift speed then becomes 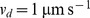. For *V. alginolyticus*, we obtain the larger value 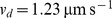. For smaller values with 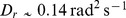, the chemotactic drift speed of *V. alginolyticus* becomes smaller than that of *E. coli*; as in the above case, for sufficiently large 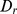, the winner of the chemotaxis race is the run-reverse-flick swimmer *V. alginolyticus*, see Fig. 6. However, as the swimming speed of *V. alginolyticus* 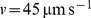 is more than twice that of *E. coli* 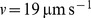, we also compare the chemotactic index, defined by 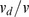. For 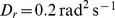 or smaller values, 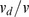 for *E. coli* (5.3%) is almost twice as large as for *V. alginolyticus* (2.7%). In this sense, the relative chemotaxis race trophy goes to *E. coli*. Ref. [19] reports the experimental observation that “*V. alginolyticus* has a threefold larger chemotactic velocity than *E. coli*.” Our analytical results clearly show that the motility pattern alone cannot explain the threefold difference in the chemotactic behavior observed experimentally. In fact, the only unknown in our model is the response function of *V. alginolyticus* bacteria, which for the sake of comparison we set to be the same as of *E. coli*. It is therefore natural to conclude that a different response function of *V. alginolyticus* is the key to interpret the experimental data of Ref. [16].

## Discussion

We have demonstrated how the careful analysis of bacterial motility patterns could quantitatively describe the dispersal of cells in homogeneous environments and the chemotactic drift velocity in small gradients of signaling chemicals. When the characteristic length scale on which the chemical concentration changes is much larger than the average run length of the cell, it is possible to use a continuous description for the density of cells 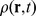. Its dynamics can be described by an advection-diffusion equation, as part of the Keller-Segel model for chemotactic aggregation [50], where the drift term represents the effect of chemotaxis and biases the otherwise uniform spreading of cells,
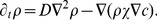
(29)Here, 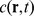 denotes the chemical field and 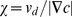 is defined as the chemotactic sensitivity and assumed to be constant. We can consider an oversimplified setting of an infinite one-dimensional domain with an imposed gradient of the chemical and find a stationary solution for this problem. One can show that the density of cells follows the profile of the chemical and has the following shape:
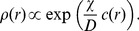
(30)This stationary profile depends on the ratio of the chemotactic drift coefficient 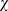 and diffusion constant 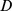. If we estimate this ratio for *E. coli* and *V. alginolyticus*, again assuming the same response function, they appear to be remarkably close to each other: 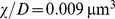 (*E. coli*), 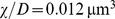 (*V. alginolyticus*). This result cannot be directly compared to the available experimental data [16], where gradients are very steep and the characteristic width of the cloud of cells around the source of the chemoattractant becomes comparable to the average length of the cell run. Nevertheless, the significantly different extensions of the cell clumps forming around the source hint to a difference in the response function between the two bacterial species (and not the motility pattern) as the reason for the observed behavior.

It appears that many cells, which are able to perform chemotaxis, have the motility patterns discussed in this paper. In particular, our analytical approach is not limited to the bacterial world, but can also be applied to swimming algae [51], cells moving during the early stages of embryo development [52], or artificial nano swimmers [53] — all of them demonstrating a very similar motility pattern. There are some examples, like swimming *P. putida* bacteria, which in addition to a reversal have also two different speeds for backward and forward motion [54]. With some minor modifications such a scenario can easily be incorporated into the framework developed here. The fact that the motility pattern of cells is now accounted for rigorously, makes it possible to apply the model to the existing data on drift velocities or agglomeration experiments [8], [16]. This way, it is feasible to access the characteristics of cells, like the response function, which would require much more sophisticated experiments to be measured directly. The response functions of various bacteria might have different functional forms or different strengths and depend on the chemical nature of the signal. We believe that our theoretical framework, complemented by numerical simulations, may serve as an excellent tool to test various hypotheses regarding the response of bacteria and check their consistency with experimental data for various motility patterns of bacteria observed in nature and laboratory.

## Supporting Information

Text S1 **How the motility pattern of bacteria affects their dispersal and chemotaxis.**
(PDF)
