# Correction: How the Motility Pattern of Bacteria Affects Their Dispersal and Chemotaxis

**DOI:** 10.1371/journal.pone.0092348

**Published:** 2014-03-24

**Authors:** The PLOS ONE Staff

Several errors were introduced in the preparation of this article for publication.

In the third sentence of the "Random walk with one turning angle" section of the Results, a symbol was removed from the definition of the Fourier-Laplace-transform. The correct definition can be found here:


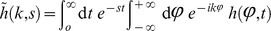


In the second sentence of the second paragraph of the "Comparison of the chemotactic drift speed for *E. coli* and *V. alginolyticus*" section of the Results, a symbol was removed from the Rotational diffusion value. The correct value can be found here:


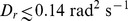


The publisher apologizes for these errors.
