# Supplementary material for: How the Motility Pattern of Bacteria Affects Their Dispersal and Chemotaxis
Source: PLoS One. 2013 Dec 31;8(12):e81936. doi: 10.1371/journal.pone.0081936 (PMC3876982; doi:10.1371/journal.pone.0081936)
Supplement: Text S1 — How the motility pattern of bacteria affects their dispersal and chemotaxis. (PDF) [file pone.0081936.s001.pdf]

How the motility pattern of bacteria affects their dispersal and chemotaxis (Supporting Information)

Johannes Taktikos[1,2,3*], Holger Stark[2], Vasily Zaburdaev[1,3]
1 Max-Planck-Institut für Physik komplexer Systeme, Dresden, Germany (current affiliation)
2 Technische Universität Berlin, Institut für Theoretische Physik, Berlin, Germany
3 Harvard University, School of Engineering and Applied Sciences, Cambridge, MA, United States
∗ E-mail: taktikos@pks.mpg.de

I. Details of the continuous time random walk model

To find the joint probability density $P(\varphi_1, t_1; \Delta\varphi, t_2)$, we use the result for the two-point PDF calculated for continuous time random walks (CTRW) in Refs. [33–35]. In Fourier-Laplace space, it becomes

$$\widetilde{P}(k_1, p; k_2, s) = \frac{\hat{F}(p)\hat{F}(s)}{[1-\hat{f}(p)\bar{g}(k_1)][1-\hat{f}(s)\bar{g}(k_2)]} + \frac{1-\bar{g}(k_2)}{1-\hat{f}(s)\bar{g}(k_2)}\,\frac{\mathcal{L}[f(t_1+\Delta t)](p,s)-\hat{F}(p)\hat{F}(s)}{1-\hat{f}(p)\bar{g}(k_1)}. \tag{S1}$$

Eq. (10) directly follows for $k_1 = 0$, $k_2 = 1$, and the jump distribution $g(\varphi)$ from Eq. (3). For the exponential run time distribution, this expression simplifies dramatically and yields

$$\widetilde{P}(k_1 = 0, p; k_2 = 1, s) = \frac{\tau_{\rm run}}{p\,[1-\cos\varphi_0 + s\tau_{\rm run}]}. \tag{S2}$$

For the case of the power law distributed run times from Eq. (17), we proceed with the asymptotic analysis, assuming t_1, $t_2 \gg \tau_0$, meaning s, $p \to 0$. The leading terms in this expansion after the inverse Laplace transform yield Eq. (18).

In the following, we derive our main result for the velocity autocorrelation function, MSD, and diffusion coefficient of the run-reverse-flick pattern. As defined in Eq. (19), we denote the probability density function of turning angles at reversals and flicks by $g_r(\varphi)$ and $g_f(\varphi)$, respectively. The probability densities of run times after reversals and flicks are denoted as $f_r(\tau)$ and $f_f(\tau)$, respectively. The corresponding survival probabilities are $F_{r,f}(\tau) = 1 - \int_0^\tau \mathrm{d}t\, f_{r,f}(t)$. At this stage, we consider a CTRW in the angle $\varphi(t)$ without rotational diffusion during the run events; due to Eq. (6), the latter can be included in the velocity autocorrelation function afterwards. To formulate the CTRW, we introduce the auxiliary probability densities $\nu_{r,f}(\varphi, t)$, with $\nu_{r,f}(\varphi, t)\mathrm{d}\varphi\,\mathrm{d}t$ denoting the probability that a reversal (r) or flick (f) occurs during the time interval $[t, t+\mathrm{d}t]$, while the cell moves along the direction $[\varphi, \varphi+\mathrm{d}\varphi]$. We also refer to $\nu_{r,f}(\varphi, t)$ as the frequency of reversals and flicks. Similar to the standard CTRW model, these densities satisfy the integral equations (see Ref. [33] for example)

$$\nu_f(\varphi, t) = \int_{-\infty}^{+\infty}\mathrm{d}\psi \int_0^t \mathrm{d}\tau\, f_r(\tau) g_r(\psi)\nu_r(t-\tau, \varphi-\psi) + \int_0^\infty \mathrm{d}\tau\, \frac{N_r^0(\varphi,\tau) f_r(t+\tau)}{F_r(\tau)}, \tag{S3a}$$

$$\nu_r(\varphi, t) = \int_{-\infty}^{+\infty}\mathrm{d}\psi \int_0^t \mathrm{d}\tau\, f_f(\tau) g_f(\psi)\nu_f(t-\tau, \varphi-\psi) + \int_0^\infty \mathrm{d}\tau\, \frac{N_f^0(\varphi,\tau) f_f(t+\tau)}{F_f(\tau)}, \tag{S3b}$$

where $N^0_{r,f}(\varphi, \tau)$ is the initial condition for the probability density that a cell has direction φ at time t_1 after the reversal (flick) occured time τ ago. The probability densities $P_{r,f}(\varphi, t)$ for a cell to be in the run mode after a reversal or flick with direction φ at time t is then expressed as

$$P_{r,f}(\varphi, t) = \int_0^\infty \mathrm{d}\tau\, N_{r,f}(\varphi, t, \tau), \tag{S4}$$

and $P(\varphi,t)=P_r(\varphi,t)+P_f(\varphi,t)$ gives the total probability density that a cell has direction φ at time t.

To complete the set of CTRW equations, we couple the densities $P_{r,f}(\varphi,t)$ to the frequencies $\nu_{r,f}(\varphi,t)$ via [33]

$$P_r(\varphi,t) = \int_{-\infty}^{+\infty} \mathrm{d}\psi \int_0^t \mathrm{d}\tau\, F_r(\tau) g_r(\psi)\nu_r(t-\tau,\varphi-\psi) + \int_0^\infty \mathrm{d}\tau\, \frac{N_r^0(\varphi,\tau)F_r(t+\tau)}{F_r(\tau)}, \quad \text{(S5a)}$$

$$P_f(\varphi,t) = \int_{-\infty}^{+\infty} \mathrm{d}\psi \int_0^t \mathrm{d}\tau\, F_f(\tau) g_f(\psi)\nu_f(t-\tau,\varphi-\psi) + \int_0^\infty \mathrm{d}\tau\, \frac{N_f^0(\varphi,\tau)F_f(t+\tau)}{F_f(\tau)}. \quad \text{(S5b)}$$

The initial condition $N^0_{r,f}(\varphi,\tau)$ is of most general form. For our purpose of calculating $P(\varphi_1,t_1;\Delta\varphi,t_2)$, it will be given as a result of the previous evolution during the time t_1. In that case, also $t_2=t_1+t$ and $\varphi=\Delta\varphi$. We introduce the notation

$$P^0_{r,f}(\varphi,\tau) = \frac{N^0_{r,f}(\varphi,\tau)F_{r,f}(t+\tau)}{F_{r,f}(t)}, \quad \text{(S6a)}$$

$$\nu^0_{r,f}(\varphi,\tau) = \frac{N^0_{r,f}(\varphi,\tau)f_{r,f}(t+\tau)}{F_{r,f}(t)}, \quad \text{(S6b)}$$

and after a Fourier-Laplace transform and some algebraic manipulations, we obtain

$$\widetilde{\nu}_f(k,s) = \frac{\hat{f}_r(s)\bar{g}_r(k)\widetilde{\nu}^0_r(k,s)+\widetilde{\nu}^0_f(k,s)}{1-\hat{f}_r(s)\hat{f}_f(s)\bar{g}_r(k)\bar{g}_f(k)}, \quad \text{(S7a)}$$

$$\widetilde{\nu}_r(k,s) = \frac{\hat{f}_f(s)\bar{g}_f(k)\widetilde{\nu}^0_f(k,s)+\widetilde{\nu}^0_r(k,s)}{1-\hat{f}_r(s)\hat{f}_f(s)\bar{g}_r(k)\bar{g}_f(k)}. \quad \text{(S7b)}$$

For the densities $\widetilde{P}_{r,f}(k,s)$, we find

$$\widetilde{P}_f(k,s) = \bar{P}^0_f(k)\hat{F}_f(s) + \bar{g}_f(k)\hat{F}_f(s)\,\frac{\hat{f}_r(s)\bar{g}_r(k)\widetilde{\nu}^0_r(k,s)+\widetilde{\nu}^0_f(k,s)}{1-\hat{f}_r(s)\hat{f}_f(s)\bar{g}_r(k)\bar{g}_f(k)}, \quad \text{(S8a)}$$

$$\widetilde{P}_r(k,s) = \bar{P}^0_r(k)\hat{F}_r(s) + \bar{g}_r(k)\hat{F}_r(s)\,\frac{\hat{f}_f(s)\bar{g}_f(k)\widetilde{\nu}^0_f(k,s)+\widetilde{\nu}^0_r(k,s)}{1-\hat{f}_r(s)\hat{f}_f(s)\bar{g}_r(k)\bar{g}_f(k)}. \quad \text{(S8b)}$$

To proceed, we have to take into account the correct initial conditions (previous evolution during t_1). We restrict to the simpler and experimentally relevant case of exponential run time distributions,

$$f_{r,f}(\tau) = \frac{1}{\tau_{r,f}}\exp\left(-\frac{\tau}{\tau_{r,f}}\right), \qquad \tau_r,\ \tau_f > 0. \quad \text{(S9)}$$

Now, we have to find the time evolution during t_1. Although we can implement an arbitrary initial condition at $t=-t_1$, we will proceed in a different way. It is easy to show that

$$P^{0,\,\mathrm{eq}}_{r,f} = \frac{\tau_{r,f}}{\tau_r+\tau_f} \quad \text{(S10)}$$

is the steady state density and for $t\in[-t_1,0]$, $P_{f,r}(k_1=0,t)=P^{0,\,\mathrm{eq}}_{f,r}=\text{const}$. Therefore, we use the following, now intermediate conditions,

$$\hat{\nu}^0_r(s) = P^{0,\,\mathrm{eq}}_f \hat{f}_f(s), \quad \text{(S11a)}$$

$$\hat{\nu}^0_f(s) = P^{0,\,\mathrm{eq}}_r \hat{f}_r(s), \quad \text{(S11b)}$$

$$P^0_{f,r} = P^{0,\,\mathrm{eq}}_{f,r}, \quad \text{(S11c)}$$

and insert them into Eq. (S8). This gives

$$\widetilde{P}(k_1=0,p;k_2,s)=\widetilde{P}_r+\widetilde{P}_f=\frac{1}{p}\,\frac{\hat{F}_r(s)\left(\hat{f}_f(s)\bar{g}_r(k_2)\tau_f+\tau_r\right)+\hat{F}_f(s)\left(\hat{f}_r(s)\bar{g}_f(k_2)\tau_r+\tau_f\right)}{(\tau_f+\tau_r)\left(1-\hat{f}_r(s)\hat{f}_f(s)\bar{g}_r(k_2)\bar{g}_f(k_2)\right)}. \tag{S12}$$

Finally, we consider explictly the turning angle distributions, $\bar{g}_{r,f}(k_2)=\cos(k_2\varphi_{r,f})$, which gives $\bar{g}_r(k_2=1)=-1$ and $\bar{g}_f(k_2=1)=0$. Using Eq. (S9), it follows that $\hat{f}_{r,f}(s)=\frac{1}{1+s\tau_{r,f}}$ and $\hat{F}_{r,f}(s)=\frac{\tau_{r,f}}{1+s\tau_{r,f}}$, and we finally arrive at

$$\widetilde{P}(k_1=0,p;k_2=1,s)=\frac{1}{p}\,\frac{\tau_r^2-\tau_f\tau_r(1-s\tau_r)+\tau_f^2(1+s\tau_r)}{(1+s\tau_f)(1+s\tau_r)(\tau_f+\tau_r)}. \tag{S13}$$

If we combine Eq. (S13) with Eqs. (8,9), we recover the velocity autocorrelation function from Eq. (20). Subsequent double-integration in time provides the MSD

$$\begin{aligned}\left\langle[\mathbf{r}(t)-\mathbf{r}(0)]^2\right\rangle_{\mathrm{rw}} \;=\; &\frac{2v^2}{(\tau_f+\tau_r)(\tau_f-\tau_r)}\Big(\tau_f^3(\tau_f-2\tau_r)e^{-t/\tau_f}-\tau_r^3(\tau_r-2\tau_f)e^{-t/\tau_r}\\ &+\left[t(\tau_f^2-\tau_f\tau_r+\tau_r^2)-(\tau_f-\tau_r)^2(\tau_f+\tau_r)\right](\tau_f-\tau_r)\Big).\end{aligned} \tag{S14}$$

The MSD displays linear scaling for large times with the diffusion coefficient

$$D=\frac{v^2}{3}\,\frac{\tau_r^3+\tau_f^3}{(\tau_r+\tau_f)^2}. \tag{S15}$$

After including rotational diffusion as well, the diffusion coefficient becomes the expression from Eq. (24).

II. Estimate of the strength of the chemotactic response

Here, we present an estimate for the strength of the chemotactic response W from Eq. (26). We refer to recent measurements by Vuppula *et al.* [24] for the chemotactic drift speed v_d of *E. coli* in response to the amino acid serine. In the range of experimentally accessible small gradients with $|\nabla c| = 10^{-3}\dots10^{-2}\,\mu\mathrm{M}\,\mu\mathrm{m}^{-1} = 0.6\dots6\,\mu\mathrm{m}^{-4}$, v_d is estimated as $v_d = 0.5\dots1.5\,\mu\mathrm{m\,s^{-1}}$. Thus, we choose a small gradient $|\nabla c| = 1\,\mu\mathrm{m}^{-4}$ and set $v_d = 1\,\mu\mathrm{m\,s^{-1}}$. Further, we use $v = 19\,\mu\mathrm{m\,s^{-1}}$, $\alpha = 0.33$, $\lambda = 1\,\mathrm{s}^{-1}$, and $D_r = 0.2\,\mathrm{s}^{-1}$ [13,24,42,43]. Solving the result for v_d from Eq. (27) [47] for W yields the chemotactic strength $W = 0.0458\,\mu\mathrm{m}^3$.

III. Alternative derivation of the velocity autocorrelation function

We now present a different derivation for the velocity autocorrelation function for run-reverse-flick motion, which is valid for two and three spatial dimensions. In this context, we consider a more general random walk with two alternating tumbling events, as depicted in Fig. S1. The angular changes during tumbling are specified by the preferential turning angles $\Delta\varphi_1$ and $\Delta\varphi_2$ and expressed in terms of the persistence parameters $\alpha = \langle\cos\Delta\varphi_1\rangle$ and $\beta = \langle\cos\Delta\varphi_2\rangle$. It turns out that only the mean cosines of the turning angles, α and β, enter the directional correlation function and the corresponding diffusion coefficient. Note that different turning angle distributions can yield the same persistence parameter. However, in the exact limiting cases of α, $\beta = \pm1$, only delta-peaked distributions at angles 0° and 180° are possible.

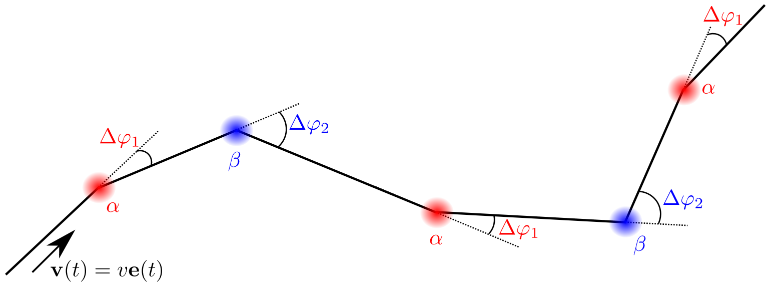

Figure S1. Pattern of the random walk with two alternating tumbling events. The persistence parameters $\alpha = \langle\cos\Delta\varphi_1\rangle$ and $\beta = \langle\cos\Delta\varphi_2\rangle$ correspond to the two preferential turning angles $\Delta\varphi_1$ and $\Delta\varphi_2$, respectively. The walker moves with constant speed v along the direction $\mathbf{e}(t)$, and the run times are exponentially distributed with mean τ_{run}.

Our goal is to determine the directional part of the velocity autocorrelation function, $C_{\text{rw}}(t,t') = v^2 \langle\mathbf{e}(t)\cdot\mathbf{e}(t')\rangle_{\text{rw}}$. We first neglect rotational diffusion during the run events and assume an exponential run time distribution $f(\tau) = \lambda\exp(-\lambda\tau)$, where λ is the tumbling rate and the mean run time is $\tau_{\text{run}} = \lambda^{-1}$. The survival probability that a run that starts at $t = 0$ is not interrupted before time t is given by $F(t) = 1 - \int_0^t \mathrm{d}\tau\, f(\tau) = \exp(-\lambda t)$. For a random walk whose first turning event is specified by α, the directional correlation function is given by

$$\begin{aligned}\langle\mathbf{e}(t)\cdot\mathbf{e}(0)\rangle_{\text{rw}} \quad &= \quad F(t) + \int_0^t \mathrm{d}t_1\, f(t_1)\alpha F(t-t_1) + \int_0^t \mathrm{d}t_2 \int_0^{t_2} \mathrm{d}t_1\, f(t_1)\alpha f(t_2-t_1)\beta F(t-t_2) \\ &\quad + \int_0^t \mathrm{d}t_3 \int_0^{t_3} \mathrm{d}t_2 \int_0^{t_2} \mathrm{d}t_1\, f(t_1)\alpha f(t_2-t_1)\beta f(t_3-t_2)\alpha F(t-t_3) + \ldots \end{aligned} \tag{S16}$$

The interpretation of the terms on the right-hand side of Eq. (S16) is as follows. A run may start at $t = 0$ and is not interrupted till t, such that the direction of motion is constant; a run may also take place from time $0\ldots t_1$, then tumbling of type α occurs, and a second run without tumbling follows during $t_1\ldots t$; the next term considers the possibility of a run $(0\ldots t_1)$, tumbling of type α at t_1, another run $(t_1\ldots t_2)$, tumbling of type β at t_2, and one more run up to time t, etc. The emergence of products $\alpha^{N_\alpha}\beta^{N_\beta}$ in the higher-order terms of Eq. (S16) stems from the occurrence of N_α and N_β tumbling events of type α and β, respectively. It is important to note that Eq. (S16) is valid in two and three dimensions.

To calculate the series in Eq. (S16), we observe that for the exponential distribution $f(t_1)f(t_2-t_1)\times\ldots\times f(t_n-t_{n-1})F(t-t_n) = \lambda^n e^{-\lambda t}$. Using

$$\int_0^t \mathrm{d}t_n \int_0^{t_n} \mathrm{d}t_{n-1}\cdots\int_0^{t_2} \mathrm{d}t_1 = \frac{t^n}{n!}, \tag{S17}$$

Eq. (S16) can be written as

$$\langle\mathbf{e}(t)\cdot\mathbf{e}(0)\rangle_{\text{rw}} = e^{-\lambda t}\left[1 + \alpha\,\frac{\lambda t}{1!} + \alpha\beta\,\frac{(\lambda t)^2}{2!} + \alpha^2\beta\,\frac{(\lambda t)^3}{3!} + \alpha^2\beta^2\,\frac{(\lambda t)^4}{4!} + \ldots\right], \tag{S18}$$

where the time-dependent terms $e^{-\lambda t}\,\frac{(\lambda t)^k}{k!}$ coincide with the Poisson distribution. Using series expansions for hyperbolic functions results in the expression

$$\langle\mathbf{e}(t)\cdot\mathbf{e}(0)\rangle_{\text{rw}} \quad = \quad e^{-\lambda t}\left[\sqrt{\frac{\alpha}{\beta}}\,\sinh\left(\sqrt{\alpha\beta}\,\lambda t\right) + \cosh\left(\sqrt{\alpha\beta}\,\lambda t\right)\right]. \tag{S19}$$

Eq. (S19) is not symmetric with respect to interchanging $\alpha \leftrightarrow \beta$. This reflects the fact that, so far, each random walker interrupts the first run with a tumbling event of type α. However, we are interested in modeling an ensemble of walkers where the first tumbling event is either of type α or β with the same probability. To generalize Eq. (S19) to an ensemble average for arbitrary times, we thus take into account that, when choosing a walker of the ensemble, the probability is 1/2 for the next tumbling event to be α or β. Formally, we symmetrize Eq. (S19) with respect to α and β, and obtain

$$\langle \mathbf{e}(t) \cdot \mathbf{e}(t') \rangle_{\mathrm{rw}} = e^{-\lambda|t-t'|} \left[\frac{\alpha+\beta}{2\sqrt{\alpha\beta}} \sinh\left(\sqrt{\alpha\beta}\,\lambda|t-t'|\right) + \cosh\left(\sqrt{\alpha\beta}\,\lambda|t-t'|\right) \right]. \tag{S20}$$

Next, we generalize the result for $\langle \mathbf{e}(t) \cdot \mathbf{e}(t') \rangle$ if we also take into account rotational diffusion during the runs. Rotational diffusion in three dimensions with rotational diffusion constant D_r leads to $\langle \mathbf{e}(t) \cdot \mathbf{e}(t') \rangle_{\mathrm{rot}} = \exp(-2D_r|t-t'|)$. With rotational diffusion, Eq. (S16) is modified according to

$$\begin{aligned} \langle \mathbf{e}(t) \cdot \mathbf{e}(0) \rangle \;=\;& F(t)e^{-2D_r t} + \int_0^t \mathrm{d}t_1\, f(t_1) e^{-2D_r t_1} \alpha F(t-t_1) e^{-2D_r(t-t_1)} \\ &+ \int_0^t \mathrm{d}t_2 \int_0^{t_2} \mathrm{d}t_1\, f(t_1) e^{-2D_r t_1} \alpha f(t_2-t_1) e^{-2D_r(t_2-t_1)} \beta F(t-t_2) e^{-2D_r(t-t_2)} + \ldots \end{aligned} \tag{S21}$$

Compared to Eq. (S16), Eq. (S21) contains an additional factor of $\exp(-2D_r t)$. Note that this statement also holds true if the run times are not exponentially distributed; it corresponds to the factorization according to Eq. (7). The MSD is given by

$$\begin{aligned} \left\langle [\mathbf{r}(t)-\mathbf{r}(0)]^2 \right\rangle \;=\;& \frac{v^2}{[(1-\alpha\beta)\lambda^2 + 4D_r(\lambda+D_r)]^2} \Bigg[\big[(1-\alpha\beta)\lambda^2 + 4D_r(\lambda+D_r)\big]\big[(2+\alpha+\beta)\lambda + 4D_r\big]t \\ &+ \Bigg\{ \frac{\lambda^2\big[\alpha+\beta+\alpha\beta(4+\alpha+\beta)\big] + 4D_r\lambda(\alpha+\beta+2\alpha\beta) + 4D_r^2(\alpha+\beta)}{\sqrt{\alpha\beta}} \sinh(\sqrt{\alpha\beta}\lambda t) \\ &\quad + 2\big[\lambda(1+\alpha)+2D_r\big]\big[\lambda(1+\beta)+2D_r\big]\cosh(\sqrt{\alpha\beta}\lambda t) \Bigg\} e^{-(\lambda+2D_r)t} \\ &- 2\big[\lambda(1+\alpha)+2D_r\big]\big[\lambda(1+\beta)+2D_r\big] \Bigg], \end{aligned} \tag{S22}$$

from which we deduce the diffusion coefficient

$$D = \lim_{t\to\infty} \frac{\left\langle [\mathbf{r}(t)-\mathbf{r}(0)]^2 \right\rangle}{6t} = \frac{v^2}{6} \frac{(2+\alpha+\beta)\lambda + 4D_r}{(1-\alpha\beta)\lambda^2 + 4D_r(\lambda+D_r)}. \tag{S23}$$

Setting $\alpha = -1$ and $\beta = 0$ yields the diffusion coefficient for run-reverse-flick motion from Eq. (23); setting $\alpha = \beta$ provides Eq. (14). Recall that this derivation, based on Eq. (S21), is independent of the spatial dimension. In addition, it does not depend on the details of the distribution of turning angles $\Delta\varphi_1$ and $\Delta\varphi_2$, but only on the persistence parameters α and β.

IV. Calculation of the chemotactic drift speed for run-tumble-flick motion

Here, we calculate the chemotactic drift speed v_d for the motility pattern run-tumble-flick. This is the random walk where tumbling events, specified by persistence parameter α, alternate with flicks, where a new direction of motion is randomly chosen. In the random walk scheme of Fig. S1, this corresponds to setting $\beta = 0$. For *V. alginolyticus*, the reversal between forward and backward runs results in $\alpha = -1$.

The run times are exponentially distributed, and for simplicity, we assume a single mean run time $\tau_{\text{run}} = \lambda^{-1}$ for forward and backward motion in a homogeneous environment. We consider a small chemical gradient $|\nabla c|$ in z direction, and the concentration $c(t)$ at the cell's position $z(t)$ becomes

$$c(t) = |\nabla c|\, z(t). \tag{S24}$$

Next, we generalize de Gennes' approach from Eq. (25) for the time-dependent rates of forward and backward runs,

$$\lambda_i(t) = \lambda \left(1 - \int_{-\infty}^{t} \mathrm{d}t'\, c(t') R(t-t')\right), \qquad i = r,\, b \tag{S25}$$

where the index r denotes the forward run after the flick, and b the backward run after the reversal. Note that we assume the same chemotactic response function after both kinds of turning event; a more general calculation can be found in Ref. [52]. The adaptive response, $\int_0^\infty \mathrm{d}t\; R(t) = 0$, has allowed us to set a possible constant concentration in Eq. (S24) to zero, as it does not affect the tumbling rates in Eq. (S25). We will use the experimentally measured response function $R(t)$ of *E. coli* [Eq. (26)], as we are primarily interested in understanding the influence of the random walk pattern on the chemotactic drift speed.

To simplify forthcoming calculations, we make use of the structure of Eq. (S25) and first consider the special case of a delta-response in time $R(t) = A\,\delta(t-T)$ with delay time T and strength A, as it was done in Refs. [47, 48]. We denote the chemotactic drift speed for the delta-response as v_δ. Having found this intermediate result for v_δ, it is generalized to the chemotactic drift speed v_d by integrating v_δ with the response function $R(t)$, according to

$$v_d = \int_0^\infty \mathrm{d}T\; R(T)\, \frac{v_\delta(T)}{A}. \tag{S26}$$

We calculate v_δ for a random walk with a flick at $t = 0$. The flick totally randomizes the direction of the subsequent forward run, which is interrupted at time t_r. At t_r, the direction of motion is changed with persistence parameter α and the particle performs a backward run of duration t_b. As every flick event destroys the directional persistence and memory of the previous random walk steps, v_δ is calculated by considering the mean displacement along the z axis during one cycle of the run-tumble-flick process. To obtain v_δ in a first-order expansion of $|\nabla c|$, we determine the average displacement of a forward run $\langle z_r\rangle$ and a subsequent backward run $\langle z_b\rangle$. As these expressions are first order in $|\nabla c|$, the mean duration of the cycle is given by $2\tau_{\text{run}}$, and the chemotactic drift speed for the delta-response becomes

$$v_\delta = \frac{\langle z_r\rangle + \langle z_b\rangle}{2\tau_{\text{run}}}. \tag{S27}$$

The random walker starts with a flick at $t = 0$ from the initial position $z = 0$. We need to calculate the displacement along the direction of the gradient, the z axis, during the forward run, $t \in [0, t_r]$, and the subsequent backward run, $t \in [t_r, t_r + t_b]$. The probability density $p_r(0 \to t_r)$ that a forward run starts at $t = 0$ and stops at t_r is given by

$$p_r(0 \to t_r) \;=\; -\frac{\mathrm{d}}{\mathrm{d}t_r} \exp\left(-\int_0^{t_r} \mathrm{d}t'\, \lambda_r(t')\right), \tag{S28a}$$

$$p_b(t_r \to t_r + t_b) \;=\; -\frac{\mathrm{d}}{\mathrm{d}t_b} \exp\left(-\int_{t_r}^{t_r+t_b} \mathrm{d}t'\, \lambda_b(t')\right), \tag{S28b}$$

and $p_b(t_r \to t_r + t_b)$ is the probability density that a backward run starts at t_r and stops at $t_r + t_b$. The time-dependent rates $\lambda_i(t)$ $(i = r,\, b)$ due to chemotaxis are given in Eq. (S25). Taking a delta-response $R(t) = A_i\,\delta(t - T_i)$ with strength A_i and delay time T_i, and using Eq. (S24), we can write the rates as

$$\lambda_i(t) = \lambda\left[1 - A_i |\nabla c|\, z(t - T_i)\right], \tag{S29}$$

and they thus depend on the previous positions $z(t-T_i)$.

The mean displacement $\langle z\rangle$ of the forward run $\langle z_r\rangle$ and subsequent backward run $\langle z_b\rangle$ reads

$$\langle z\rangle = \langle z_r\rangle + \langle z_b\rangle \tag{S30a}$$

$$= \left\langle \int_0^\infty \mathrm{d}t_r\, p_r(0\to t_r) z(t_r)\right\rangle + \left\langle \int_0^\infty \mathrm{d}t_r \int_0^\infty \mathrm{d}t_b\, p_r(0\to t_r) p_b(t_r\to t_r+t_b) z(t_b)\right\rangle . \tag{S30b}$$

The averages $\langle\cdots\rangle$ in Eq. (S30) are taken over all possible paths due to random swimming directions after the flick event. In addition, they represent ensemble averages to account for the rotational diffusion during the runs. Without rotational diffusion, we could write the positions in Eq. (S30b) as $z(t_i) = v_z(t_i)t_i$, where $v_z(t)$ is the z component of the velocity $\mathbf{v}(t)$. To proceed, we shift the averages $\langle\cdots\rangle$ into the integrals in Eq. (S30b).

We start to calculate $\langle z_r\rangle$:

$$\langle z_r\rangle = \int_0^\infty \mathrm{d}t_r \left\langle \left[-\frac{\mathrm{d}}{\mathrm{d}t_r}\exp\left(-\int_0^{t_r}\mathrm{d}t'\,\lambda_r(t')\right)\right] z(t_r)\right\rangle \tag{S31a}$$

$$= \int_0^\infty \mathrm{d}t_r \left\langle \exp\left(-\int_0^{t_r}\mathrm{d}t'\,\lambda_r(t')\right)\frac{\mathrm{d}z(t_r)}{\mathrm{d}t_r}\right\rangle \tag{S31b}$$

$$= \int_0^\infty \mathrm{d}t_r\, e^{-\lambda t_r}\left(\langle v_z(t_r)\rangle + \lambda A_r|\nabla c|\int_0^{t_r}\mathrm{d}t'\,\langle z(t'-T_r)v_z(t_r)\rangle\right). \tag{S31c}$$

In the first step, a partial integration led us to Eq. (S31b). Then we introduced $v_z(t_r) = \frac{\mathrm{d}z(t_r)}{\mathrm{d}t_r}$ as the speed in z direction, and substituted Eq. (S29) into (S31b); after expanding the exponential, we only kept the first-order terms in $|\nabla c|$ to arrive at Eq. (S31c). The averages in this equation will be calculated without chemotaxis to remain first-order in $|\nabla c|$. We immediately find $\langle v_z(t_r)\rangle = 0$, as the direction of motion after the flick at $t=0$ is random. Further, we use $z(t) = \int_0^t \mathrm{d}s\, v_z(s)$ and get

$$\langle z_r\rangle = |\nabla c|\, A_r \int_0^\infty \mathrm{d}t_r\,\lambda e^{-\lambda t_r}\int_0^{t_r}\mathrm{d}t'\int_0^{t'-T_r}\mathrm{d}s\,\langle v_z(s)v_z(t_r)\rangle. \tag{S32}$$

Without chemotaxis, the velocity distribution is isotropic, which implies

$$\langle v_z(s)v_z(t_r)\rangle = \frac{v^2}{3}\,\langle \mathbf{e}(s)\cdot\mathbf{e}(t_r)\rangle\,, \tag{S33}$$

and the directional correlation function reads

$$\langle \mathbf{e}(s)\cdot\mathbf{e}(t_r)\rangle = \begin{cases} e^{-2D_r(t_r-s)} & 0\le s< t_r,\\ 0\,, & \text{else.}\end{cases} \tag{S34}$$

After inserting Eqs. (S33) and (S34) into Eq. (S32), we perform the resulting integrals and obtain the mean displacement of a forward run,

$$\langle z_r\rangle = |\nabla c|\, A_r\,\frac{v^2}{3}\,\frac{e^{-(\lambda+2D_r)T_r}}{(\lambda+2D_r)^2}\,, \tag{S35}$$

which is proportional to the gradient $|\nabla c|$ and the response strength A_r. To check for consistency, we note that Eq. (S35) agrees with Locsei's intermediate result for $\alpha = 0$ from Ref. [47].

Now, we turn to the mean displacement of the backward run $\langle z_b\rangle$ from Eq. (S30). We proceed in the same way as before and obtain up to first order in $|\nabla c|$:

$$\langle z_b\rangle = \int_0^\infty \mathrm{d}t_r\int_0^\infty \mathrm{d}t_b\,\lambda e^{-\lambda t_r-\lambda t_b}\Big\{\langle v_z(t_b)\rangle - A_r\,|\nabla c|\,\langle z(t_r-T_r)v_z(t_b)\rangle$$
$$+\lambda A_r|\nabla c|\int_0^{t_r}\mathrm{d}t'\,\langle z(t'-T_r)v_z(t_b)\rangle + \lambda A_b|\nabla c|\int_{t_r}^{t_r+t_b}\mathrm{d}t'\,\langle z(t'-T_b)v_z(t_b)\rangle\Big\}. \tag{S36}$$

Again, the expectation values are calculated without chemotaxis, such that $\langle v_z(t_r)\rangle = 0$ and Eq. (S33) holds. Be aware that $v_z(t_b)$ is the speed in z direction at the absolute time $t_r + t_b$. The directional correlation function becomes

$$\langle \mathbf{e}(s)\cdot\mathbf{e}(t_b)\rangle = \begin{cases} e^{-2D_r t_b}\,\alpha\, e^{-2D_r(t_r-s)}\,, & 0 \le s < t_r,\\ e^{-2D_r(t_r+t_b-s)}\,, & t_r \le s < t_r + t_b,\\ 0\,, & \text{else.}\end{cases} \tag{S37}$$

To obtain the first line of Eq. (S37), we made the decomposition $\langle \mathbf{e}(s)\cdot\mathbf{e}(t_b)\rangle = \langle \mathbf{e}(t_b)\cdot\mathbf{e}(t_r^+)\rangle\,\langle \mathbf{e}(t_r^+)\cdot\mathbf{e}(t_r^-)\rangle\,\langle \mathbf{e}(t_r^-)\cdot\mathbf{e}(s)\rangle$, see also Ref. [47]. We obtain

$$\begin{aligned}\langle z_b\rangle \;=\; & \frac{|\nabla c|\,v^2}{3(\lambda+2D_r)^3}\Bigg\{-2\alpha A_r D_r e^{-(\lambda+2D_r)T_r}\\ & +A_b\, e^{-(\lambda+2D_r)T_b}\Big[2D_r(1+\alpha\lambda T_b)+\lambda[1+\alpha(1+\lambda T_b)]\Big]\Bigg\}.\end{aligned} \tag{S38}$$

Note that the dependence of $\langle z_b\rangle$ on A_r reflects a chemotactic coupling between forward and backward runs. Finally, we consider equal strength of the chemotactic response $A_r = A_b = A$ and $T_r = T_b = T$, and combine all preceding results according to Eq. (S27). In first-order in $|\nabla c|$, we thus arrive at the chemotactic drift speed for the delta-response,

$$v_\delta = A|\nabla c|\,v^2\lambda e^{-(\lambda+2D_r)T}\,\frac{\lambda[2+\alpha(1+\lambda T)]+2D_r[2+\alpha(\lambda T-1)]}{6(\lambda+2D_r)^3}\,. \tag{S39}$$

The final drift speed v_d from Eq. (28) is obtained by integrating v_δ with the response function from Eq. (26) according to Eq. (S26).
